# PD-1 is a marker of activation on tumor infiltrating NK cells in head and neck cancer

**DOI:** 10.1186/2051-1426-3-S2-P398

**Published:** 2015-11-04

**Authors:** Fernando Concha-Benavente, Raghvendra M Srivastava, Benjamin Kansy, Robert L Ferris

**Affiliations:** 1 University of Pittsburgh, Pittsburgh, PA, USA; 2 University of Pittsburgh Cancer Institute, Pittsburgh, PA, USA; 3 Department of Otorhinolaryngology, University Hospital Essen, Essen, Germany

## 

Co-inhibitory immune checkpoint receptors have become important targets for cancer immunotherapy. Programmed death 1 (PD-1) has been well-characterized on T cells in many cancer types, including head and neck cancer (HNC), for its ability to mediate activation and eventually T cell exhaustion in the tumor microenvironment. However, PD-1 expression on NK cells, which are crucial innate immune effector cells against cancer, remains largely undefined. In the setting of HNC, NK cells mediate lysis of EGFR-overexpressing tumor targets via cetuximab-mediated antibody dependent cytotoxicity (ADCC). Indeed, cetuximab has shown to be clinically effective but only to a modest extent. Therefore, it is necessary to investigate how cetuximab modulates activation of immune effector cell infiltrates in the tumor microenvironment in order to improve or extend its therapeutic efficacy. We hypothesized that expression of PD-1 *per se* on NK cells may constitute a marker of a chronically activated phenotype, which is suppressed only after ligation by its cognate ligand programmed death ligand-1 (PD-L1). Thus, tumor cell-expressing PD-L1 may present as a crucial mediator of immunosuppression in the tumor microenvironment decreasing cytotoxicity of cetuximab activated PD-1 expressing NK cells. Herein, using The Cancer Genome Atlas (TCGA) data for 500 HNC patients' tumors, we found that PD-1 expression correlates with NK activation markers. Indeed, HNC patients also exhibit higher levels of circulating and tumor infiltrating PD-1^+^ NK cells, and neoadjuvant cetuximab treatment increased this frequency *in vitro* and *in vivo* in a prospective Phase II trial. In addition, anti-PD-1 mAb nivolumab enhanced cetuximab mediated NK cell activation and HNC cell lysis. Therefore, blocking PD-L1/PD-1 axis may be a useful approach to reverse immune evasion of HNC tumors to cetuximab therapy by reversing NK cell dysfunction.

